# Global research trends on bacterial contamination and microbiological quality of ready-to-eat foods: a bibliometric analysis

**DOI:** 10.3389/frma.2025.1719169

**Published:** 2026-01-21

**Authors:** Kawooya Abubaker, Emmanuel Eilu, Saheed Adekunle Akinola, Hussein Mukasa Kafeero, Makeri Danladi, Ismail Abiola Adebayo, Jesca Nakavuma

**Affiliations:** 1 Department of Microbiology and Immunology, Kampala International University, Bushenyi, Uganda; 2 Institute of Allied Health Sciences, Clarke International University, Kampala, Uganda; 3 Department of Microbiology and Parasitology, College of Medicine and Health Sciences, School of Medicine and Pharmacy, University of Rwanda, Butare, Rwanda; 4 Department of Medical Microbiology, Faculty of Health Sciences, Habib Medical School, Islamic University in Uganda, Mbale, Uganda; 5 Department of Microbiology, Parasitology, and Immunology, School of Medicine, Kabale University, Kabale, Uganda; 6 College of Veterinary Medicine, Animal Resources and Biosecurity, Makerere University, Kampala, Uganda

**Keywords:** antimicrobial resistance, bacterial contamination, bibliometric analysis, food safety, global health, ready-to-eat foods

## Abstract

**Background:**

Ready-to-eat (RTE) foods are increasingly consumed worldwide due to urbanization, dietary shifts, and globalization of food systems, yet they remain a significant vehicle for foodborne diseases. Despite the growing body of research, a systematic mapping of global scientific trends in this area has been lacking.

**Methods:**

We conducted a bibliometric analysis of RTE food microbiology research indexed in Scopus from 1973 to 2025. Publication trends, citation patterns, leading authors, institutions, journals, and country-level contributions were assessed using Bibliometrix. Network analyses (co-authorship, bibliographic coupling, and keyword co-occurrence) were performed using VOSviewer to identify collaborative structures and thematic evolution.

**Results:**

A total of 780 publications across 256 sources were identified, growing at an average annual rate of 6.9%. The field accumulated 19,811 citations, with highly cited works between 1996 and 2015 establishing its intellectual foundations. China, Italy, and the USA led in productivity, while Public Health England, Universidad de Córdoba, and China's Institute of Microbiology were major institutional hubs. Journal of Food Protection, International Journal of Food Microbiology, and Food Control dominated as key sources. Collaboration networks revealed strong international linkages, particularly among high-income countries. Keyword analysis showed two thematic axes: persistent focus on classical pathogens *(Listeria monocytogenes, Salmonella, Bacillus cereus*) and emerging concerns related to convenience foods, and fast foods.

**Conclusion:**

Research on the microbiological safety of RTE foods has grown steadily, reflecting global recognition of its public health significance. However, outputs remain concentrated in high-income countries, while low- and middle-income regions with high foodborne disease burdens are underrepresented. Future research should prioritize equitable global participation, integration of genomic and omics tools, and translation of findings into food safety policy. This bibliometric evidence highlights the need for stronger international collaboration to ensure the microbiological safety of RTE foods in an era of rapid dietary transition.

## Introduction

Ready-to-eat (RTE) foods, defined as foods that can be consumed without further preparation, cooking, or reheating, have become an integral part of modern diets worldwide (Choudhury et al., 2021; Cui et al., 2024). Urbanization, the expansion of food-service industries, longer working hours, and shifting consumer preferences toward convenience have all driven a marked increase in the production, distribution, and consumption of RTE products, from salads and sandwiches to street-vended snacks and packaged chilled meals (de Bruin et al., 2021). While these foods provide important benefits, time saving, dietary variety and expanded access to nutrition, they also create pathways for microbial contamination at multiple points along the farm-to-fork continuum (Bhatia et al., 2024; Founou et al., 2021; Alegbeleye et al., 2018). Because RTE foods are often consumed without a final kill-step, any microbial contamination introduced during production, handling, storage, or distribution can persist into the food as consumed and pose direct risks to consumers (Amsalie, 2021; Olaimat and Holley, 2012).

Bacterial contamination of RTE foods is a leading cause of foodborne illness globally. A wide range of bacterial pathogens and indicator organisms *Salmonella* spp., *Listeria monocytogenes, Staphylococcus aureus, Escherichia coli, Bacillus cereus* and a host of spoilage and opportunistic bacteria, have been detected in RTE items across settings (Klištincová et al., 2024; Tao et al., 2022; Bintsis, 2017). Depending on pathogen identity, contamination level, host susceptibility and dose, outcomes may range from self-limited gastroenteritis to severe invasive disease, and in vulnerable populations can result in hospitalization or death (Herranz-Ulldemolins et al., 2025; Fleckenstein et al., 2021; Mirriam et al., 2013). Beyond acute infections, poor microbiological quality undermines food security, generates economic losses through recalls and waste, and erodes consumer confidence in food systems (Alberto and Gonzalez, 2024; Gizaw, 2019; Hussain and Dawson, 2013).

Over the past decades a robust body of empirical research has examined the prevalence, determinants and control of bacterial contamination in RTE foods (Berezhanskiy et al., 2025; Churchill et al., 2019; Mengistu et al., 2022). Studies span laboratory-based microbiological surveys, outbreak investigations, risk assessments, and evaluations of hygiene interventions in industrial, retail and informal food sectors. However, the growth of literature in this area is heterogenous across regions and disciplines: microbiologists, food technologists, public health practitioners, food safety regulators and social scientists have contributed distinct but overlapping perspectives. This multidisciplinary nature complicates synthesis and obscures broader trends in where, how and by whom RTE microbiological quality is being studied.

Bibliometric analysis, a quantitative assessment of scientific literature using publication metadata, citation patterns and text-mining, offers a complementary lens to traditional systematic reviews (Fu et al., 2023). Rather than focusing on the findings of individual studies, bibliometrics maps the structure of a research field: publication trajectories over time, geographic and institutional distributions, collaboration networks, influential journals and authors, thematic clusters, and emergent topics. For public health and food safety, such a bird's-eye view can reveal critical gaps, patterns of cross-disciplinary collaboration or isolation, and the diffusion of methods and concepts. Importantly, bibliometric methods help stakeholders; research funders, policy-makers, practitioners prioritize research investment, target capacity building, and align surveillance efforts with evidence needs (Ding, 2025). Despite the policy and practice relevance of RTE food safety, there is currently no comprehensive, global bibliometric synthesis that specifically maps research on bacterial contamination and microbiological quality of RTE foods. Existing narrative or systematic reviews tend to focus on specific pathogens, food categories, or geographic regions (Berezhanskiy et al., 2025; Abdul and Pavoni, 2025), but do not systematically chart the global research outlook. Without such a synthesis, it is difficult to assess whether research output aligns with public health burden, which regions or food types are under-studied, and how methodological innovation is shaping the field.

This bibliometric analysis addresses these gaps by providing a structured, data-driven overview of global research trends on bacterial contamination and microbiological quality of RTE foods. The study aims to: (1) assess publication and citation trends over time, (2) identify leading contributors, countries, institutions, authors, and journals, (3) map collaboration and citation networks, and (4) detect thematic evolution through keyword analysis. By providing a structured overview of research activity, this work highlights progress to date and points to areas requiring future attention to strengthen food safety and public health globally.

## Methods

For this bibliometric analysis, data were retrieved from the Scopus database on 16 September 2025. The search strategy targeted publications addressing bacterial contamination and the microbiological quality of ready-to-eat (RTE) foods. Search terms were selected to balance sensitivity and specificity and were developed by combining key concepts relevant to the study using Boolean operators (“OR,” “AND”) and truncation (“^*^”). Terms related to ready-to-eat foods (“ready to eat,” “ready-to-eat,” and “RTE”) were included to capture the most commonly used descriptors of this food category across scientific and regulatory literature, while both hyphenated and non-hyphenated forms were used to maximize retrieval. The term food was included to restrict the search to the food safety domain and avoid unrelated environmental or clinical studies.

Keywords related to bacterial contamination and microbiological quality (e.g., “bacteria,” “bacterial,” “microbiological,” “foodborne pathogen^*^,” and “food contamination”) were selected to encompass both pathogen-specific investigations and broader microbiological assessments commonly reported in food microbiology research. The final search strategy, applied to titles and abstracts to enhance reproducibility while maintaining thematic relevance, was: TITLE-ABS [(“ready to eat” OR “ready-to-eat” OR “RTE”) AND food AND (bacteria OR bacterial OR microbiological OR “foodborne pathogen” OR “food contamination”)]. No additional restrictions were applied at the search stage.

This strategy retrieved 2,009 Scopus-indexed records. All authors independently screened the retrieved records based on predefined inclusion criteria, namely original research articles reporting primary microbiological isolation of bacteria from ready-to-eat foods. A total of 1,229 records that did not meet these criteria were excluded, resulting in 780 publications included in the final bibliometric analysis. A PRISMA-style flow diagram summarizing the identification, screening, and inclusion process is provided as Supplementary File 1.

### Data cleaning and standardization

Author names, institutional affiliations, and country information were standardized prior to analysis to ensure accurate attribution and reproducibility. Bibliographic records exported from Scopus were processed using Bibliometrix, which applies algorithmic disambiguation of author names based on initials, surnames, and affiliation metadata. Institutional names were reviewed and harmonized to account for variations in spelling and naming conventions (e.g., abbreviations or language differences). Country-level attribution was based on author affiliation data provided in Scopus records. Publications involving authors from multiple countries were assigned to each contributing country using a full counting approach, consistent with common bibliometric practice; therefore, a single publication may be counted toward multiple countries. International co-authorship was identified automatically through shared authorship across different country affiliations and visualized using co-authorship networks in VOSviewer. Publications with multiple institutional affiliations were retained without exclusion, and all affiliated institutions and countries were included in the analysis.

### Data analysis

The bibliographic data retrieved from Scopus were exported in BibTeX format and imported into Mendeley desktop for deduplication after which the data was exported in RIS format and analyzed using Bibliometrix (an R package) and VOSviewer version 1.6.20 (van Eck and Waltman, 2014). Bibliometrix was employed for descriptive and performance analyses, including annual publication trends, most productive journals, countries, institutions, and authors, as well as citation and collaboration metrics. The software's biblioshiny interface was further utilized to generate summary statistics and visualization outputs. For science mapping, VOSviewer was applied to construct and visualize bibliometric networks. Co-authorship (authors, institutions, countries), co-occurrence of keywords, and citation networks were generated to highlight collaboration patterns, thematic clusters, and research hotspots. The combination of Bibliometrix and VOSviewer provided both quantitative insights and graphical representations, ensuring a comprehensive mapping of global research trends on bacterial contamination and microbiological quality of ready-to-eat foods.

## Results

### Main information

From 1973 to 2025, a total of 780 publications on bacterial contamination and microbiological quality of ready-to-eat foods were identified across 256 sources, with research output growing at an average annual rate of 6.9%. The field is marked by considerable scholarly impact, with an average of 25 citations per document, and demonstrates strong collaboration, as reflected by nearly five co-authors per paper and an international co-authorship rate of 19.7%. Most outputs were original research articles (678).

### Annual scientific production

**Figure 1:**
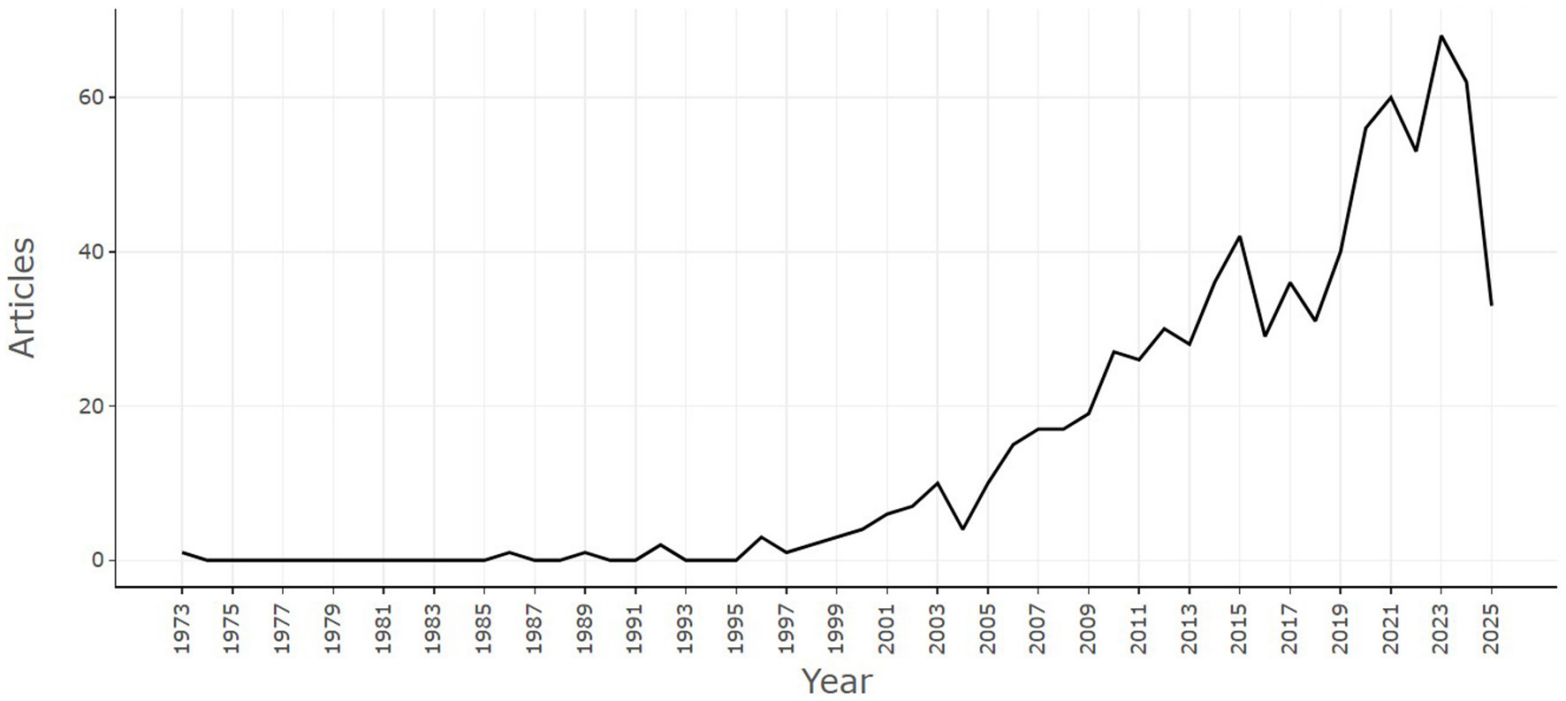
Total number of publications for year.

The earliest Scopus-indexed article on bacterial contamination and microbiological quality of ready-to-eat (RTE) foods appeared in 1973, but research activity remained negligible for more than two decades, with only sporadic publications through the mid-1990s. A gradual rise began in the late 1990s, gaining momentum in the 2000s as annual outputs surpassed 10 publications and stabilized at 15–19 papers per year by 2006–2009. The field expanded further in the 2010s, increasing from 27 articles in 2010 to 42 in 2015, signaling broader recognition of RTE food safety as a public health concern. The most rapid growth occurred in the last decade, coinciding with global attention to foodborne pathogens, antimicrobial resistance, and food system vulnerabilities. Publications peaked at 68 articles in 2023, the highest output to date, before a slight decline in 2024 (62) and a partial count in 2025 (33). Overall, the literature shows a sustained upward trajectory, with an average annual growth rate (AGR) of 6.96%, reflecting steady expansion of the research base (Figure 1).

### Citation analysis

Across the entire dataset, a total of 19,811 citations were accumulated, with a Document Average Age of 8.62 years. This citation profile demonstrates the evolution and impact of the field over five decades. Early publications (1973–1989) received few citations, reflecting the emerging stage of research, while highly cited studies emerged in the 1990s and early 2000s, such as 1992 (105 citations), 1996 (225), 1999 (256), and 2000 (490), marking foundational contributions (Table 1). Citation activity remained strong through the 2010s, with notable peaks in 2009 (1,165), 2012 (1,167), 2013 (1,081), and 2015 (1,424), highlighting sustained scholarly interest. More recent publications, particularly in 2018 (1,333) and 2020 (1,596), reflect high visibility for research addressing contemporary issues like antimicrobial resistance and emerging foodborne pathogens. Although articles from 2023 to 2025 have lower total citations due to limited citable time, their annual citation rates indicate rapid uptake.

**Table 1:** Annual trends in publications and citations on RTE Foods.

**Year**	**Number of publications**	**Total citations**	**Mean citation per article**
1973	1	0	0.00
1986	1	1	1.00
1989	1	2	2.00
1992	2	105	52.50
1996	3	225	75.00
1997	1	0	0.00
1998	2	15	7.50
1999	3	256	85.33
2000	4	490	122.50
2001	6	264	44.00
2002	7	252	36.00
2003	10	628	62.80
2004	4	212	53.00
2005	10	408	40.80
2006	15	502.1	33.47
2007	17	525	30.88
2008	17	624.9	36.76
2009	19	1,165	61.32
2010	27	915	33.89
2011	26	888.9	34.19
2012	30	1,167	38.90
2013	28	1,081	38.61
2014	36	1,005	27.92
2015	42	1,424	33.90
2016	29	705	24.31
2017	36	991.1	27.53
2018	31	1,333	43.00
2019	40	934	23.35
2020	56	1,596	28.50
2021	60	717	11.95
2022	53	559.2	10.55
2023	68	505.2	7.43
2024	62	280.9	4.53
2025	33	33.99	1.03

### Country-level scientific contributions to RTE food research

**Figure 2:**
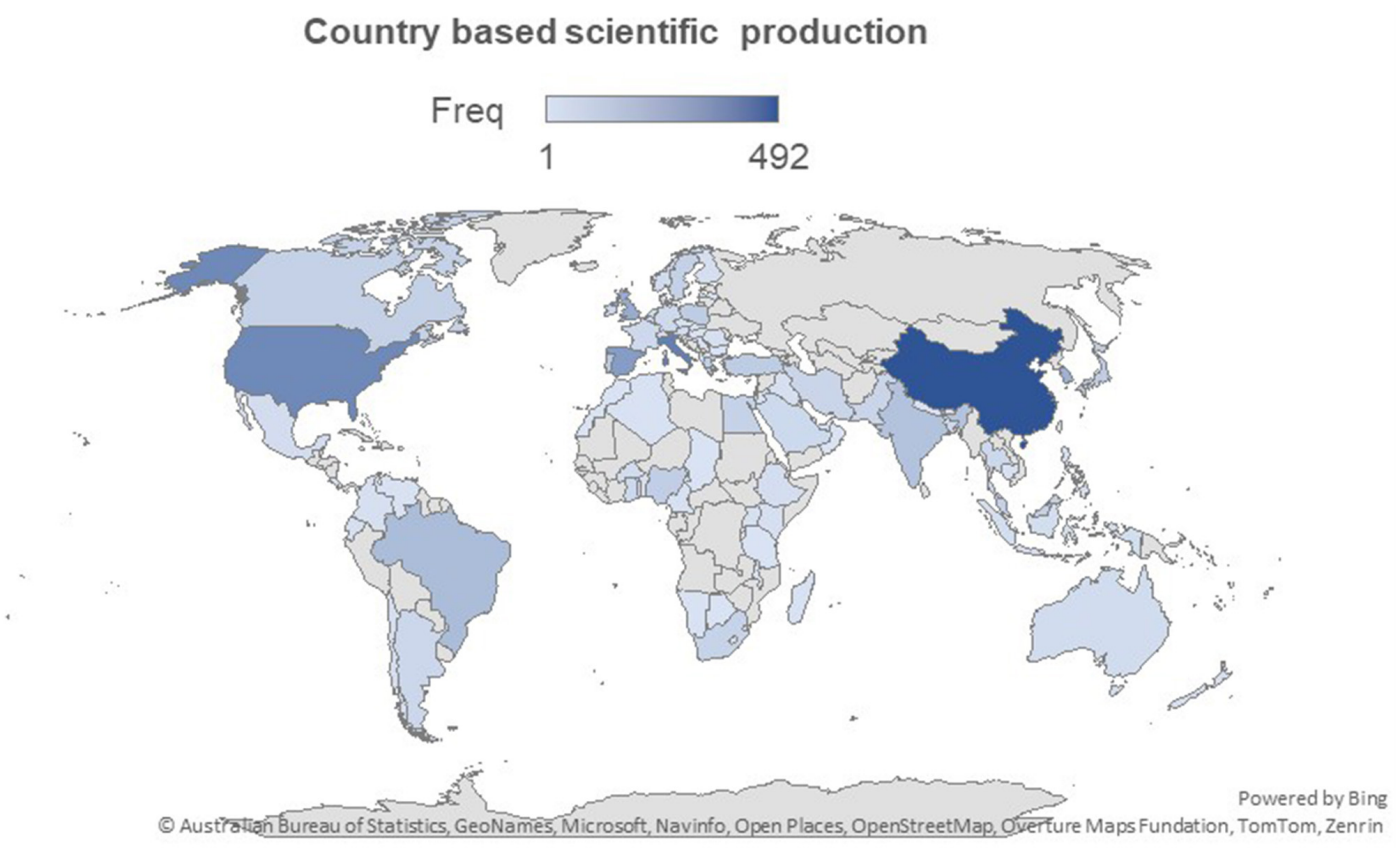
Country based scientific production (map created using Bing).

**Figure 3:**
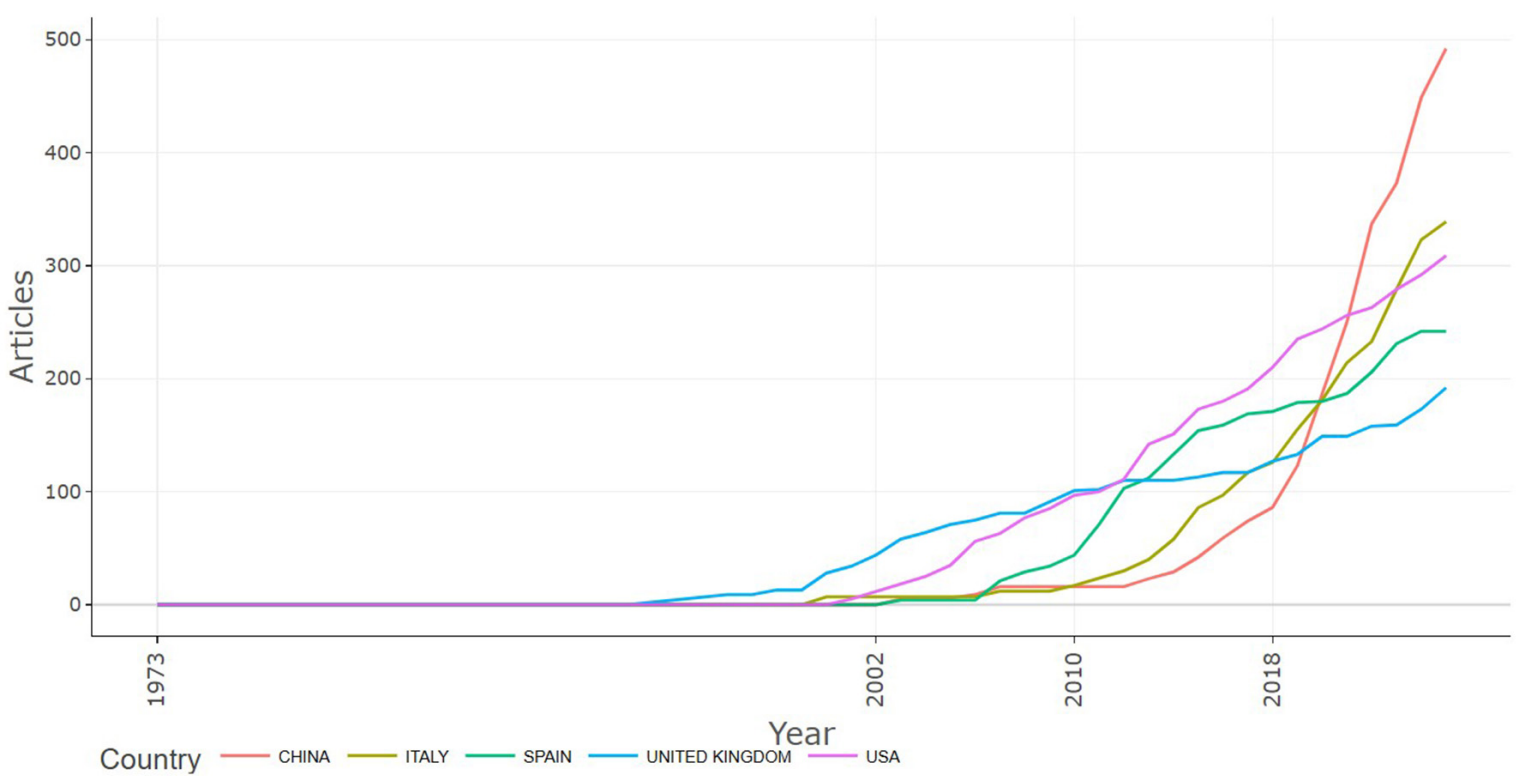
Country based scientific production over time.

The 780 publications on bacterial contamination and microbiological quality of ready-to-eat (RTE) foods originated from 83 countries (Figure 2). China is the leading contributor, accounting for 492 publications (63.1%), followed by Italy (339; 43.5%) and the United States (309; 39.6%). Other top contributors include Spain (242; 31.0%), the United Kingdom (192; 24.6%), South Korea (172; 22.1%), Brazil (133; 17.0%), and India (113; 14.5%), while Portugal (93; 11.9%) and Turkey (82; 10.5%) complete the top 10 (Table 2). It is important to note that the sum of country-based publication contributions exceeds 780, because many publications involved international co-authorship, whereby a single article is attributed to multiple countries. This overlap, also illustrated in the Countries' Collaboration World Map (Figure 3), highlights the extensive network of international partnerships that characterize the field. For example, strong collaborative ties are observed between the USA and Korea (128 joint publications), China and Japan (138), China and Malaysia (110), and the UK and Cambodia (105). Similarly, Australia frequently partners with Canada, China, France, Germany, Malaysia, and the USA, each with over 130 co-authored publications. These collaborative networks explain why the cumulative percentages exceed 100% in the individual country output.

**Table 2:** Top 15 countries in number of publications.

**Country**	**Freq**	**APPY**
China	492	41.0
Italy	339	28.3
USA	309	25.8
Spain	242	20.2
UK	192	16.0
South Korea	172	14.3
Brazil	133	11.1
India	113	9.4
Portugal	93	7.8
Turkey	82	6.8
Poland	81	6.8
Japan	80	6.7
Nigeria	74	6.2
Greece	72	6.0
Malaysia	65	5.4

APPY, Average publication per year.

**Figure 4:**
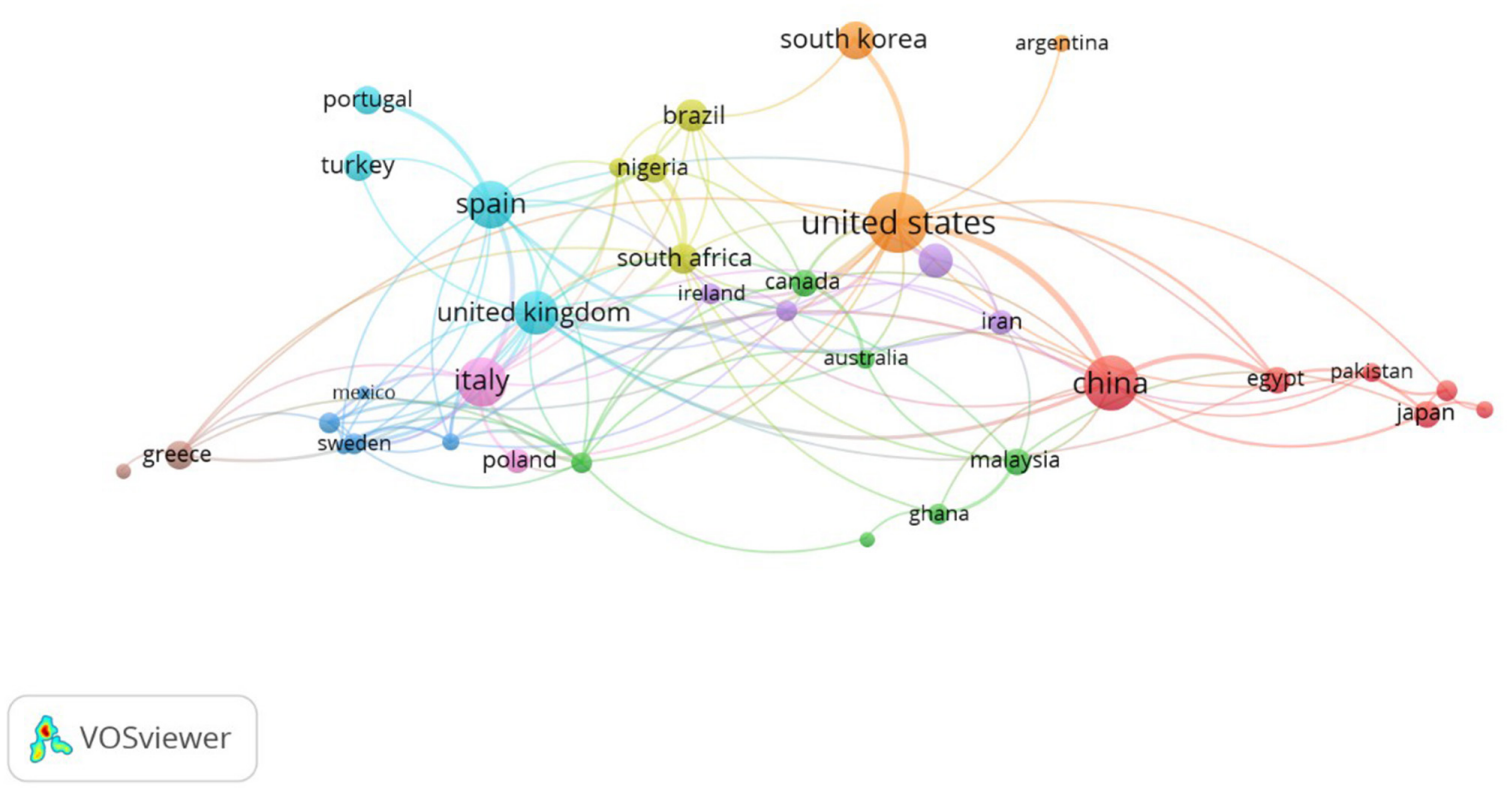
National collaboration networks.

Beyond the top 10, countries such as Poland, Japan, Nigeria, Greece, Malaysia, Canada, and Sweden also contribute significantly, demonstrating a wide geographic distribution of research activity across Asia, Europe, Africa, and the Americas. While the data may suggest that a few countries dominate scientific production, RTE food microbiology has become a globally collaborative research domain, with increasing participation from both developed and emerging countries. Analysis of countries publication trend over time (Figure 4) reveals rapid increase in scientific output from China, Italy, Spain, United Kingdom and the USA over the past two decades.

### Authorship patterns, institutional productivity, and source impact

Research on bacterial contamination and microbiological quality of ready-to-eat (RTE) foods is driven by a core group of prolific authors, institutions, and journals. Among authors, Christine L. Little leads with 15 publications and 1,133 citations, followed by Christopher H. Sommers and Robert T. Mitchell, reflecting both productivity and scholarly influence (Table 3). Institutional contributions (Table 4) are similarly concentrated, with Public Health England (UK, 71 articles), the Institute of Microbiology (China, 47), and Universidad de Córdoba (Spain, 47) at the forefront, alongside other notable organizations from the USA, Greece, Sweden, Brazil, and Italy, highlighting global engagement in the field. In terms of sources, the Journal of Food Protection dominates (77 articles, 2,890 citations), followed by the International Journal of Food Microbiology and Food Control, while journals such as Food Microbiology and Frontiers in Microbiology represent emerging influential outlets (Table 5). Together, these patterns illustrate that RTE food microbiology research is shaped by a highly interconnected network of authors, institutions, and journals, emphasizing both productivity and impact within the field.

**Table 3:** Top 10 productive authors in publishing articles on microbiological quality of RTE foods.

**Authors**	**Articles**	**Citation**
Little, Christine L.	15	1,133
Sommers, Christopher H.	11	304
Mitchell, Robert T.	9	565
Zhang, Jumei	9	354
Gillespie, Iain Andrew	8	512
Mclauchlin, J.	8	341
Valero, Antonio	8	215
Zurera-Cosano, G.	8	185
Pérez-Rodríguez, Dr. Fernando	7	132
Rhee, Min Suk	7	204

**Table 4:** Top 10 productive institutions in the field.

**Affiliation**	**Articles**	**Country**
Public Health England	71	United Kingdom
Institute of Microbiology	47	China
Universidad De Córdoba	47	Spain
Usda Ars Eastern Regional Research Center	44	USA
Agricultural University of Athens	41	Greece
Sveriges Lantbruksuniversitet	35	Sweden
Universidade De São Paulo	35	Brazil
Università Degli Studi Di Bari Aldo Moro	29	Italy
Università Degli Studi Di Milano	29	Italy
University of Shanghai for Science and Technology	28	China

**Table 5:** Leading sources on microbiological quality of RTE foods.

**Source**	**h-index**	**g-index**	**m-index**	**TC**	**NP**	**PY-Start**
Journal of Food Protection	32	52	0.941	2,890	77	1992
Food Control	29	48	1.16	2,324	52	2001
International Journal of Food Microbiology	28	50	0.824	2,548	56	1992
Food Microbiology	16	22	0.696	1,081	22	2003
Frontiers in Microbiology	15	18	1.364	845	18	2015
Journal of Food Science	12	15	0.632	330	15	2007
Foods	11	16	1.571	290	25	2019
Food Research International	10	14	0.714	227	14	2012
LWT-Food Science and Technology	10	13	0.5	303	13	2006
Foodborne Pathogens and Disease	9	14	0.563	316	14	2010

TC, Total citation; NP, Number of publication; PY-start, Publication start year.

### Cooperative network analysis

**Figure 5:**
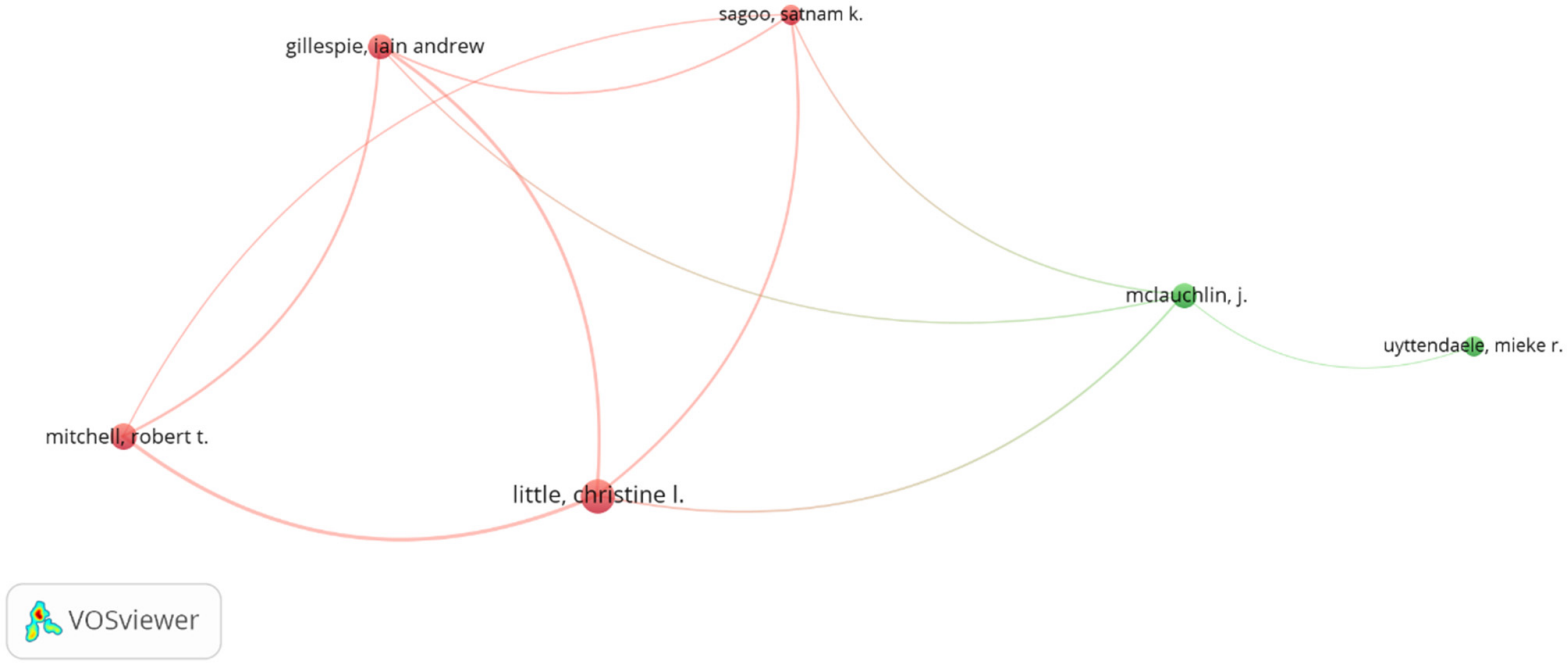
Authors collaboration networks.

Analysis of collaboration networks primarily involves analysis of collaboration between countries, institutions, and individual researchers. For this study, we utilized VOSviewer's co-authorship feature to generate a national collaborative network, with a threshold set at 5 publications and 100 citations. Thirty-seven countries met the threshold, and the total strength of the co-authorship links with other countries was calculated. Figure 3 shows countries with the highest total link strength. The analysis revealed a large collaborative network among the United States, China, the United Kingdom, Spain, Italy, and South Korea. Other countries include South Africa, Nigeria, Japan, Pakistan, Egypt, Iran, Malaysia, Poland, Mexico, Greece, Turkey, Portugal, Brazil, Argentina, Canada, Ireland, Australia, and Ghana, all with varying total link strengths. Similarly, in the author collaboration network, we set a minimum threshold of 5 documents per author with 100 citations, and 27 authors met the threshold. The analysis reveals that some of the 27 authors in the network are not connected. Figure 5 presents the largest set of connected authors.

### Bibliographic coupling

**Figure 6:**
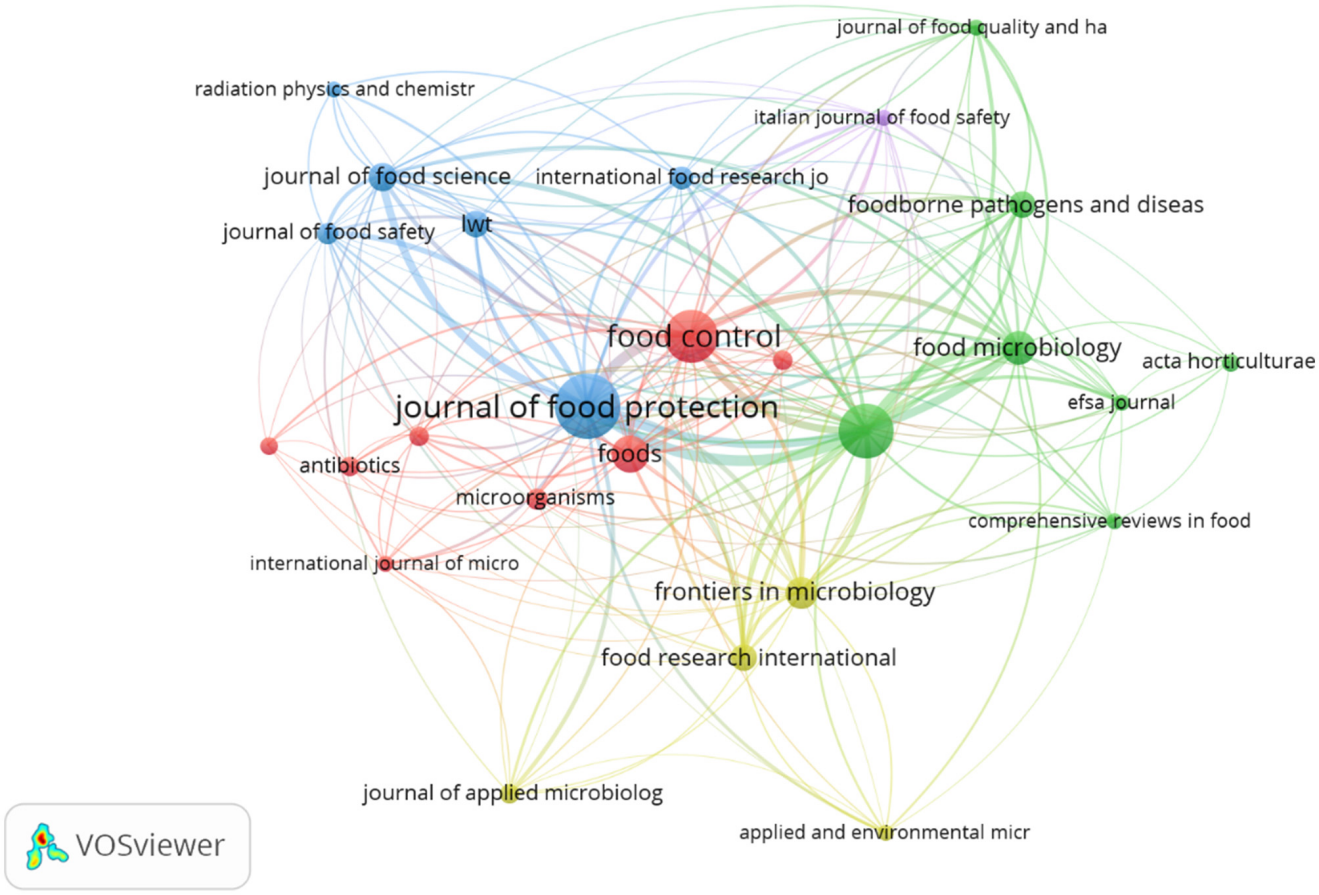
Bibliographic coupling by sources.

**Figure 7:**
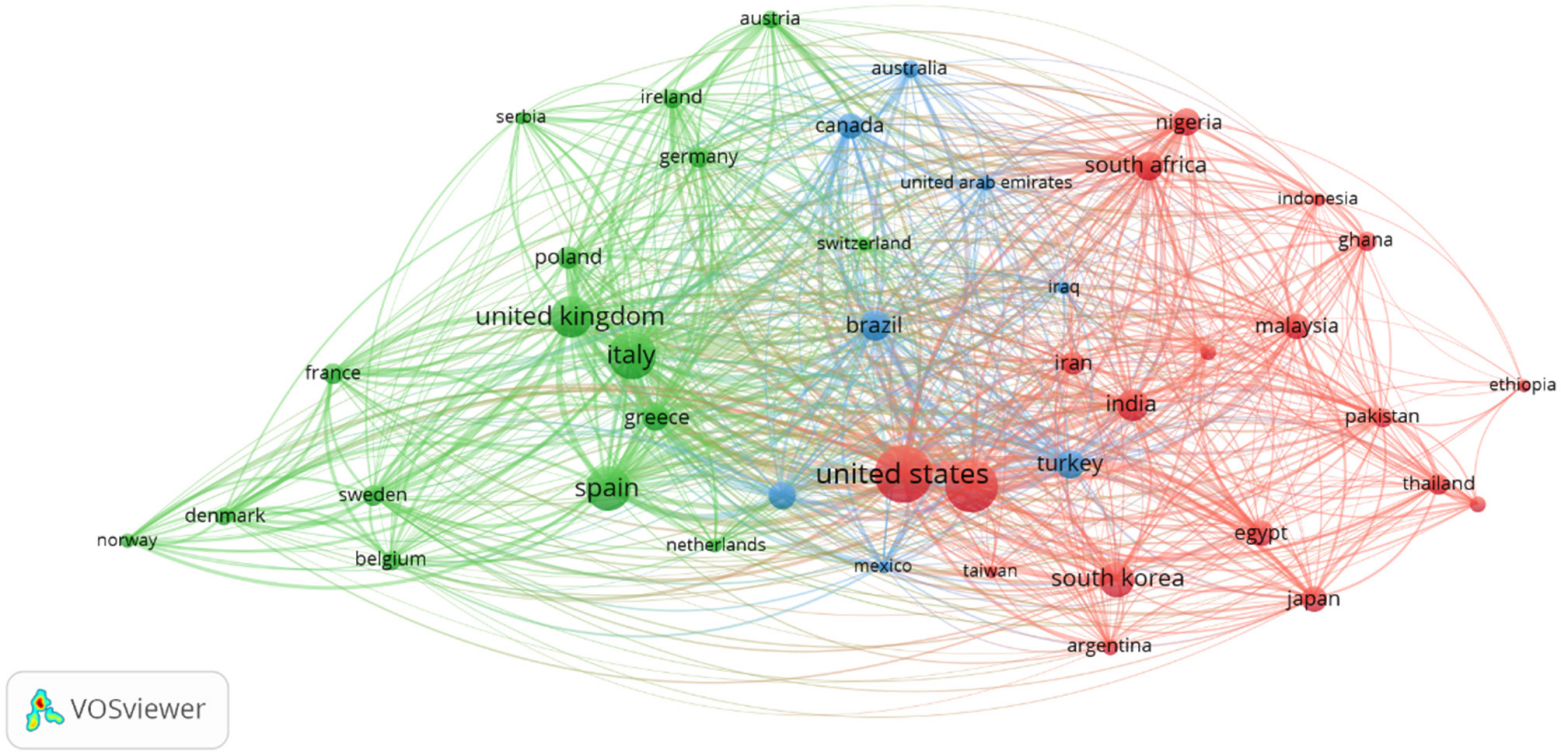
Bibliographic coupling by organizations.

**Figure 8:**
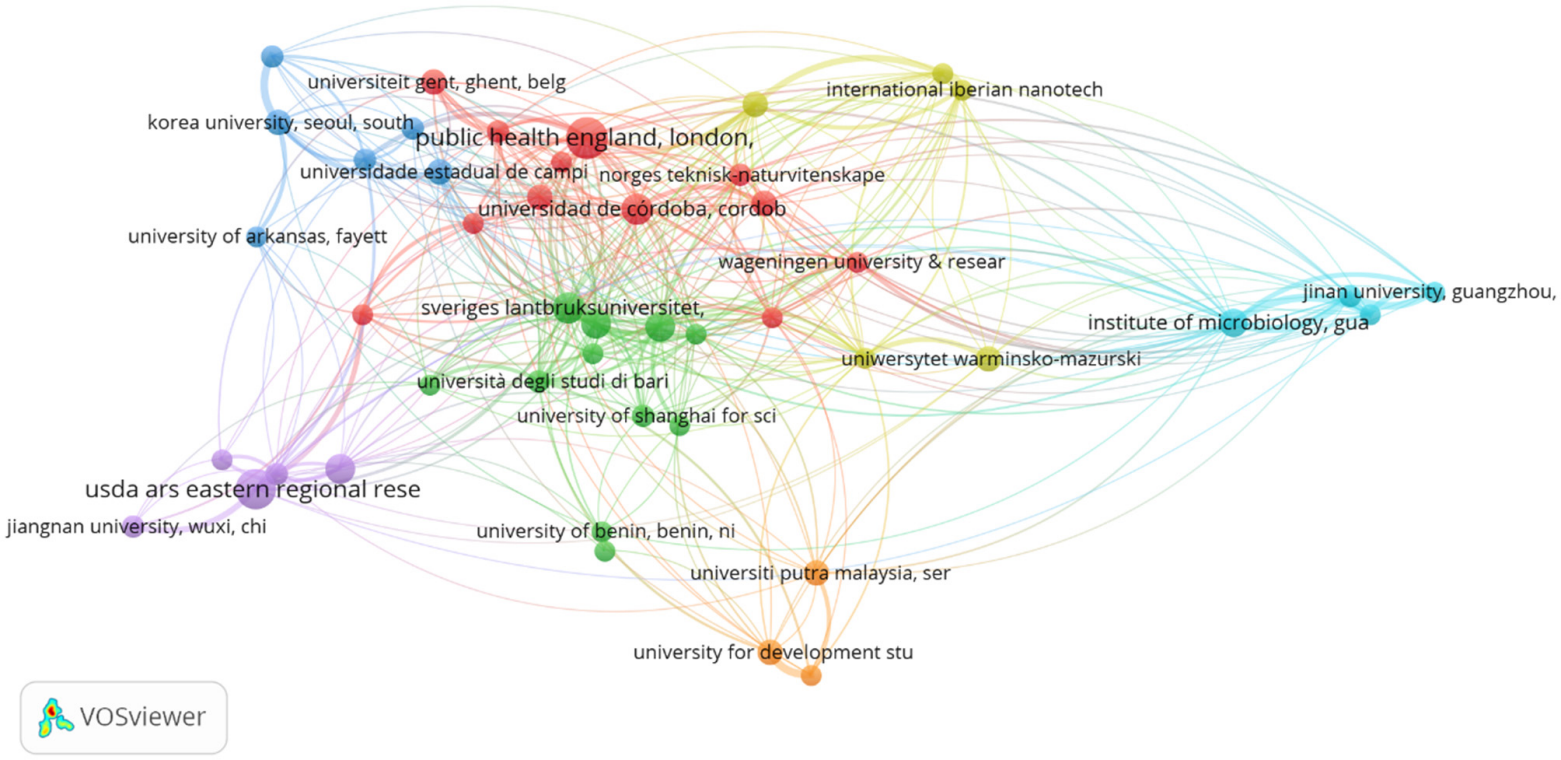
Bibliographic coupling by countries.

Bibliographic coupling was performed to explore the intellectual structure of research on bacterial contamination and microbiological quality of ready-to-eat (RTE) foods, with thresholds applied to a minimum of five documents per source or organization and a maximum of five countries per document. At the source level, 27 of 256 journals met the threshold, with the Journal of Food Protection (77 documents, 24 links, total link strength 615), International Journal of Food Microbiology (56 documents, 24 links, link strength 480), and Food Control (52 documents, 23 links, link strength 366) emerging as the most central. Among 1,003 institutions, 46 met the minimum threshold, led by Public Health England (20 documents, 24 links, link strength 112), followed by Universidad de Córdoba and Sveriges Lantbruksuniversitet (11 documents each; link strengths 61 and 80, respectively), highlighting strong institutional interconnectivity. At the country level, 43 of 97 nations met the threshold, with the USA, UK, China, Italy, and Spain forming prominent clusters, indicating both high productivity and a shared knowledge base. These findings demonstrate that RTE food research is not only concentrated in specific journals and institutions but also tightly connected internationally, reflecting both the collaborative nature of scientific output in the field (Figures 6–8).

### Keywords co-occurrence analysis

Across the 780 studies analyzed, 4,649 author keywords and 3,516 indexed keywords were identified. Keywords represent the conceptual core of scientific publications, and their co-occurrence within the same article reflects thematic relationships across the literature. To extract meaningful structures, VOSviewer's co-occurrence analysis was applied with a minimum threshold of 10 author keyword occurrences. Under this setting, 273 of the 4,649 author keywords met the inclusion criterion, and the total link strength with other keywords was calculated.

**Figure 9:**
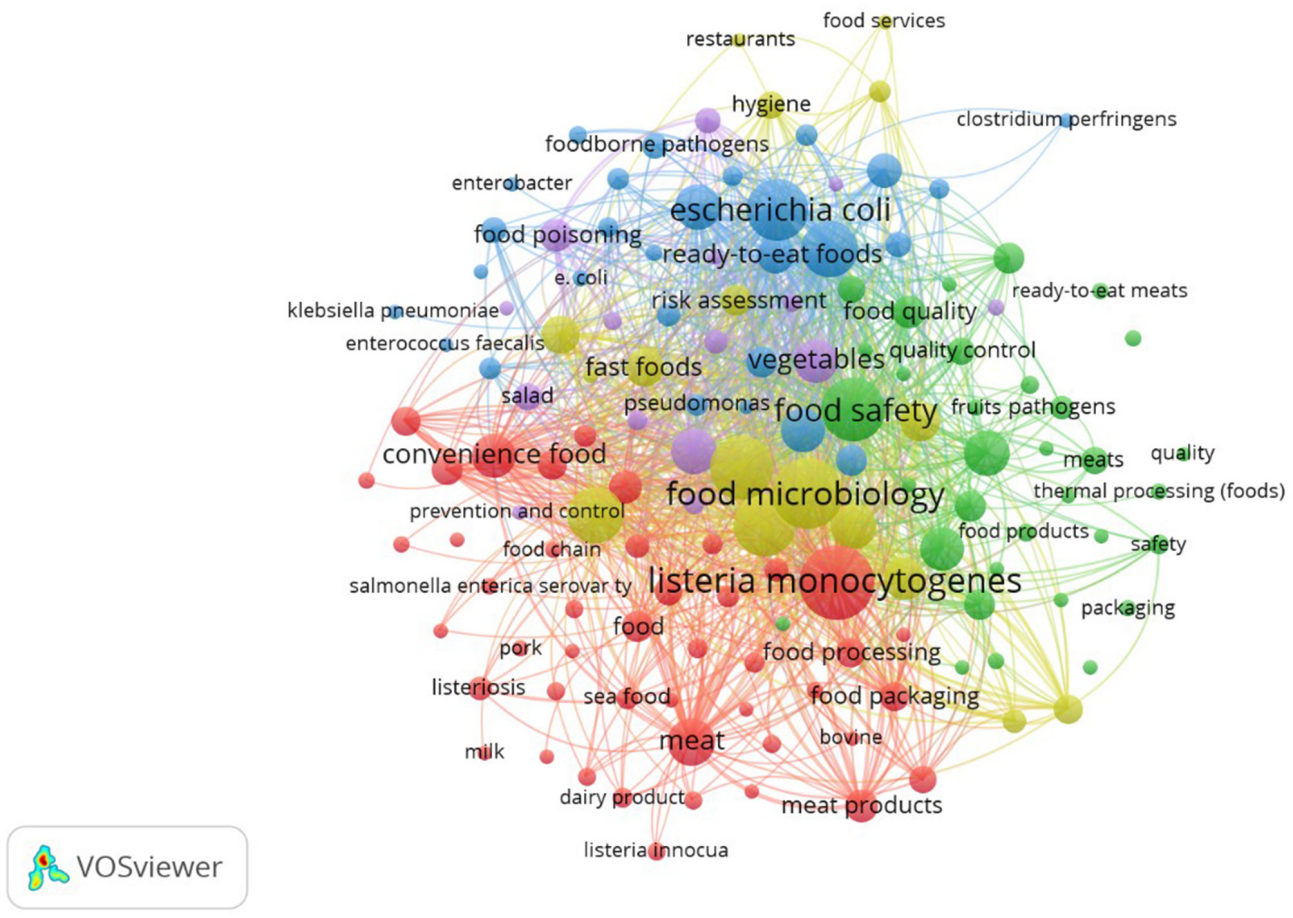
Co-occurrence network of author keywords across studies.

**Figure 10:**
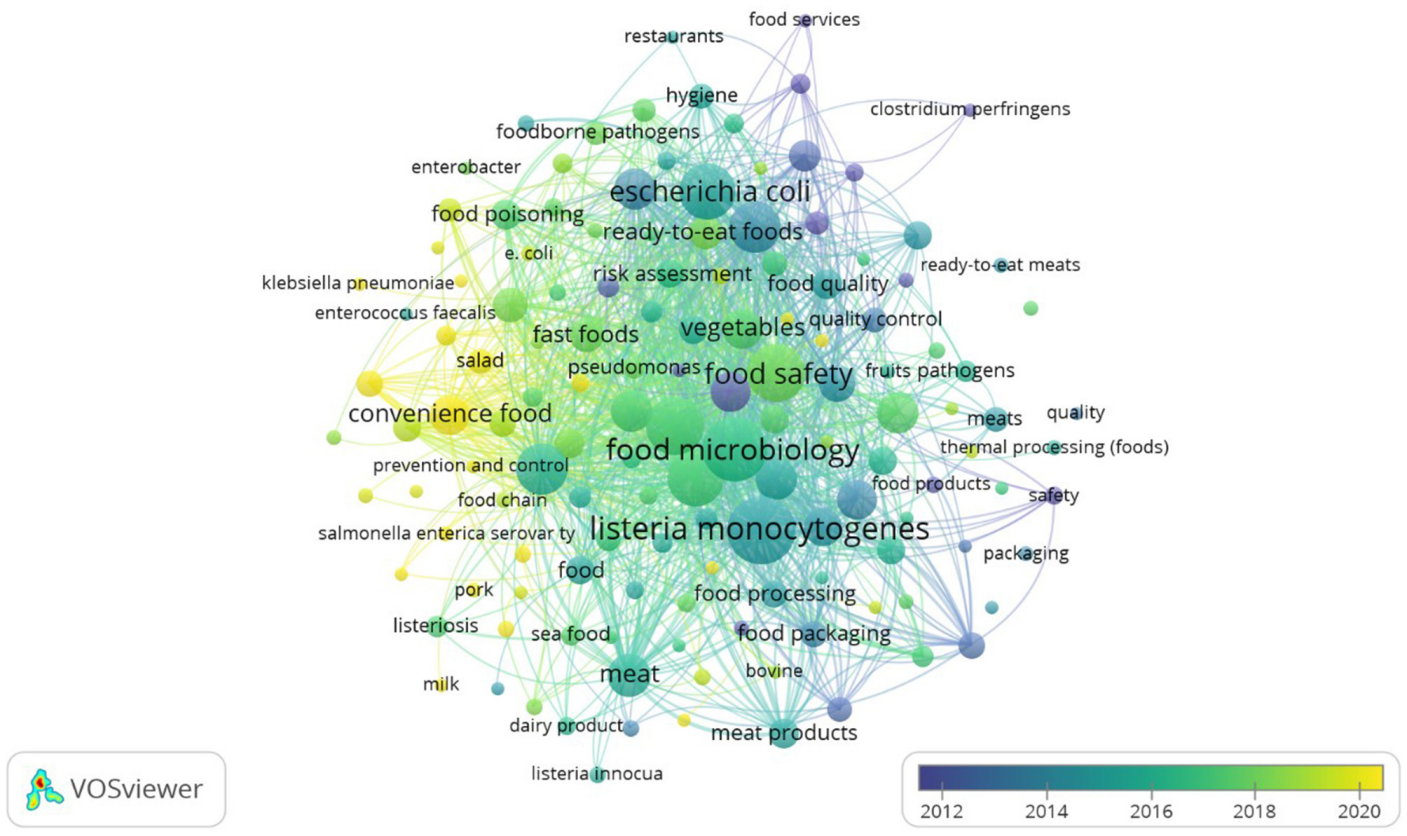
Time trend co-occurrence network of author keywords.

Figure 9 presents the author keyword co-occurrence network, where the size of each node corresponds to frequency, and the link strength represents the intensity of co-appearance with other keywords. An overlay visualization (Figure 10) further maps these keywords by average publication year and average citation count, highlighting both temporal evolution and scholarly impact. Observing Figures 9, 10 in terms of occurrence and average citations, respectively, the most frequent terms were *Listeria monocytogenes* (252; 36.13), food microbiology (234; 29.82), food control (185; 27.33), food safety (184; 26.74), and microbiology (179; 28.57 citations). These terms indicate a strong emphasis on foodborne pathogens and quality and safety frameworks. Temporal overlay analysis shows that classical pathogens such as *Bacillus cereus* (average publication year 2012), *Staphylococcus aureus* (2013), and *Salmonella* (2014) represent longstanding research areas, while emerging terms such as convenience food (2021) and fast food (2018) highlight newer concerns linked to dietary transitions and modern food systems. Citation impact analysis further underscores Salmonella (38.69 citations), Listeria monocytogenes (36.13), and Bacillus cereus (32.78) as highly influential topics, reflecting their enduring scientific and public health importance. These findings suggest that research on the microbiological quality of ready-to-eat foods is structured around two intersecting axes: (i) pathogen-centered investigations that continue to attract high scholarly attention, and (ii) emerging concerns around convenience and processed foods, which are shaping new research frontiers.

## Discussion

### Global growth of research output

Our analysis demonstrates that research on bacterial contamination and the microbiological quality of RTE foods has expanded substantially over the past five decades, with a nearly 7% annual growth rate. While activity was sparse before the mid-1990s, the surge in publications during the 2000s and beyond aligns with global recognition of foodborne pathogens as a public health priority, and increased awareness of antimicrobial resistance (AMR) as a cross-cutting threat. The marked peak in 2023 reflects not only scientific momentum but also increased surveillance and regulatory focus in response to recent food safety crises and changing dietary patterns. Similar surges have been observed in bibliometric studies of related fields, such as antimicrobial resistance and foodborne pathogens (Abdul and Pavoni, 2025; Jangid et al., 2024; Sweileh, 2021), confirming that food microbiology is increasingly embedded in global health security discourse.

The citation analysis reveals that the intellectual foundations of the field were laid during the 1990s and early 2000s, with seminal works addressing *Listeria monocytogenes, Salmonella*, and surveillance methods that continue to influence subsequent scholarship. The sustained high citation counts of studies published in 2009–2015 underscore enduring interest in classical foodborne pathogens, while the more recent prominence of 2018–2020 works reflects the rising impact of AMR, metagenomics, and molecular detection tools (Brown et al., 2019; Schadron et al., 2024; Gomes et al., 2025). Importantly, the rapid uptake of very recent publications suggests that the field remains dynamic and responsive to emerging food safety challenges.

### Geographic and institutional leadership

Research output is geographically skewed, with China, Italy, the United States, Spain, and the United Kingdom leading in productivity. This dominance reflects advanced laboratory capacity, strong food safety regulatory systems, and significant investment in food microbiology. However, the relative underrepresentation of low- and middle-income countries (LMICs), particularly in Africa, Southeast Asia, and Latin America, is concerning given the disproportionate burden of foodborne illnesses in these regions (WHO, 2025). In 2018, a World Bank study finds that the impact of unsafe food costs low- and middle-income economies about US$ 110 billion in lost productivity and medical expenses each year (World Bank Group, 2018). Encouragingly, collaboration maps reveal growing international networks, particularly involving China, the USA, the UK, and emerging economies, suggesting increasing inclusivity in knowledge production. Public Health England, Universidad de Córdoba, and China's Institute of Microbiology emerged as institutional hubs, highlighting the role of well-funded public health and academic institutions in shaping the field.

### Authorship and journal dynamics

The concentration of scholarly influence among a relatively small group of prolific authors and institutions underscores the importance of knowledge hubs in advancing this domain. Christine L. Little and collaborators, for example, have shaped the epidemiology of Listeria and Salmonella in RTE foods, providing a foundation for subsequent research. Journals such as Journal of Food Protection, Food Control, and International Journal of Food Microbiology dominate the dissemination of this work, reflecting the interdisciplinary space between applied food science and public health microbiology. The rise of newer outlets, including Frontiers in Microbiology and Foods, points to expanding opportunities for dissemination and a diversification of scholarly communication.

### Thematic evolution: pathogen-centered vs. emerging food system concerns

Keyword co-occurrence analysis highlights the dual structure of the field: pathogen-centered investigations (notably *Listeria monocytogenes, Salmonella, and Bacillus cereus*) remain dominant, while newer terms such as convenience food and fast food reflect dietary transitions and changing consumer habits. This shift aligns with the increasing global reliance on processed and ready-to-consume meals, driven by urbanization, busy lifestyles, and globalization of food systems (de Bruin et al., 2021). High citation impact associated with classical pathogens demonstrates their enduring importance, yet the growing visibility of research on AMR, street foods, and globalized supply chains indicates that the thematic focus of the field is broadening. In particular, street-vended and fast foods represent critical subcategories of ready-to-eat foods, especially in urban and low- and middle-income settings, where informal food handling, limited temperature control, and high consumer turnover increase the risk of bacterial contamination. The increasing appearance of these terms in recent years reflects growing scientific attention to their role in foodborne disease transmission and public health risk, even though research output remains uneven across regions.

### Collaboration and intellectual connectivity

The bibliographic coupling analysis reveals a tightly interconnected knowledge base, with the USA, UK, China, Italy, and Spain forming central nodes of collaboration. Strong institutional linkages suggest that RTE food microbiology is not only a mature scientific domain but also one that increasingly benefits from international cooperation. However, the fragmentation of some author networks suggests opportunities to foster broader inter-institutional and cross-regional collaborations, particularly involving LMIC researchers who are often situated in high-burden contexts but remain underrepresented in authorship networks.

### Research gaps and future directions

Despite robust growth, our findings reveal persistent research gaps. First, the dominance of high-income countries raises questions about equity and relevance, given that LMICs face the greatest risks from unsafe RTE foods sold in informal markets. Second, there is disproportionate focus on classical pathogens and limited integration of cutting-edge genomic and metagenomic surveillance in RTE food studies. Future research should therefore emphasize equitable global participation, and methodological innovation through omics approaches.

### Strengths, limitations, and implications

A key strength of this bibliometric analysis lies in its comprehensive mapping of global RTE food microbiology across five decades, providing a clear view of intellectual structures, collaboration patterns, and thematic evolution. However, limitations inherent to bibliometric methods must be acknowledged, including reliance on Scopus indexing, which underrepresents regional journals, and the citation time-lag that disadvantages very recent outputs. Despite these limitations, the analysis offers valuable insights to guide funding priorities, enhance international collaboration, and strengthen policy frameworks.

## Conclusion

This bibliometric analysis provides the first comprehensive mapping of global research on bacterial contamination and the microbiological quality of ready-to-eat foods. Over five decades, the field has demonstrated steady growth, shifting from periodic studies in the 1970s to a dynamic, interconnected research domain characterized by international collaborations. The thematic evolution highlights enduring attention to classical foodborne pathogens while also reflecting emerging concerns linked to modern dietary practices. Looking forward, future research should prioritize high-burden bacterial contaminants such as Listeria monocytogenes, Salmonella spp., and Bacillus cereus, particularly in underrepresented low- and middle-income settings. There is also a clear need to expand the application of whole-genome sequencing, metagenomics, and other omics-based approaches to strengthen surveillance, outbreak detection, and risk assessment in ready-to-eat food systems. Addressing these priorities will be critical for aligning research output with global food safety needs in the coming decade.
