# Supplementary material for: Global research trends on bacterial contamination and microbiological quality of ready-to-eat foods: a bibliometric analysis
Source: Front Res Metr Anal. 2026 Jan 21;10:1719169. doi: 10.3389/frma.2025.1719169 (PMC12868203; doi:10.3389/frma.2025.1719169)
Supplement: Supplementary file 1 [file Table_1.docx]

Records identified from:

Scopus: (n= 2009)

Records screened

(n =2009)

Duplicate records removed

(n = 0)

Titles and abstract screened

(n =2009)

Studies included

(n =780)

**Identification of studies via databases and registers**

**Identification**

**Screening**

**Included**

Records excluded after title and abstract screening

(n =1229)

PRISMA flowchart
